# Association between aspirin therapy and the outcome in critically ill patients: a nested cohort study

**DOI:** 10.1186/s40360-016-0047-z

**Published:** 2016-02-05

**Authors:** Shmeylan A. Al Harbi, Hani M. Tamim, Hasan M. Al-Dorzi, Musharaf Sadat, Yaseen M. Arabi

**Affiliations:** Pharmaceutical Care Department, College of Pharmacy, King Saud bin Abdulaziz University for Health Sciences, King Abdulaziz Medical City, Riyadh, Saudi Arabia; Department of Internal Medicine, American University of Beirut-Medical Center, Beirut, Lebanon; Intensive Care Department, College of Medicine, King Saud bin Abdulaziz University for Health Sciences, King Abdulaziz Medical City, PO Box 22490, MC 1425, Riyadh, 1426 K S A; King Abdulaziz Medical City, Riyadh, Saudi Arabia

**Keywords:** Aspirin, Outcome assessment, Intensive care, Hospital mortality, Survival, Multiple organ failure, and Propensity scores

## Abstract

**Background:**

Antiplatelet therapy may attenuate the undesirable effects of platelets on the inflammatory cascades in critical illness. The objective of this study was to evaluate the association between aspirin therapy during intensive care unit (ICU) stay and all-cause mortality.

**Methods:**

This was a nested cohort study within two randomized controlled trials in which all enrolled patients (*N* = 763) were grouped according to aspirin intake during ICU stay. The primary endpoints were all-cause ICU mortality and hospital mortality. Secondary endpoints included the development of severe sepsis during the ICU stay, ICU and hospital length of stay and the duration of mechanical ventilation. Propensity score was used to adjust for clinically and statistically relevant variables.

**Results:**

Of the 763 patients, 154 patients (20 %) received aspirin. Aspirin therapy was not associated with a reduction in ICU mortality (adjusted OR 1.18, 95 % CI 0.69–2.02, *P* = 0.55) nor with hospital mortality (adjusted OR 0.95, 95 % CI 0.61–1.50, *P* = 0.82). Aspirin use had no preferential association with mortality among any of the study subgroups. Additionally, aspirin therapy was associated with higher risk of ICU-acquired severe sepsis, and increased mechanical ventilation duration and ICU length of stay.

**Conclusion:**

Our study showed that the use of aspirin in critically ill patients was not associated with lower mortality, but rather with an increased morbidity.

**Trial Registration Number:**

ISRCTN07413772 and ISRCTN96294863.

## Background

Sepsis and multiple organ failure (MOF) are the main causes of death in intensive care units (ICUs). There is ample evidence that platelets play an important role in the progression of MOF in critically ill patients [1–5]. At cellular level, platelets have a significant influence on inflammatory processes by releasing cytokines, chemokines, and lipid mediators that have pro- or anti-inflammatory properties [6–11], activating the complement system [12, 13]. Other platelet effects include releasing antimicrobial proteins and together with neutrophils trapping bacteria [10, 14–17] and mediating adhesion to monocytes, neutrophils, and endothelial cells that lead to cellular functions such as production of cytokines, and reactive oxygen as well as recruitment and immigration of leukocytes at the site of tissue damage [7, 10, 11, 18]. Hence, the question arose whether aspirin may have a beneficial effect in critically ill patients. Several observational studies have shown that antiplatelet drugs reduce such biomarkers as C-reactive protein, soluble CD62P, and CD54, pro-inflammatory cytokines, and platelet leukocyte conjugate in these patients [19–23]. The aim of the study was to assess the association between aspirin therapy during ICU admission and all-cause mortality in a cohort of critically ill medical-surgical patients.

## Methods

### Setting

The study was a retrospective cohort study, conducted in the adult medical-surgical ICU at King Abdulaziz Medical City, a tertiary-care academic referral center in Riyadh, Saudi Arabia. The ICU admitted medical, surgical, and trauma patients, and operated as a closed unit with 24-h, 7-day onsite coverage by critical care board certified intensivists [24]. The nurse-to-patient ratio in the unit was approximately 1:1.2.

### Study design

This was a post-hoc analysis of two randomized controlled Trials (ISRCTN07413772 and ISRCTN96294863). The first trial was conducted between January 2004 and March 2006. It included 523 patients, comparing intensive insulin therapy (IIT) (for patients with a blood glucose level of 4.4–6.1 mmol/L or 80–110 mg/dl) to conventional insulin therapy (CIT) (for patients with a blood glucose level of 10–11.1 mmol/L or 180–200 mg/dl) [25]. The trial showed no significant difference in ICU mortality between the IIT and CIT groups (13.5 % vs. 17.1 %, *p* = 0.3). Hypoglycemia occurred more frequently with intensive insulin therapy (28.6 % vs. 3.1 % of patients; *p* = 0.0001) [23]. The second trial, conducted between April 2006 and January 2008, included 240 patients and assessed the effects on outcomes of permissive underfeeding (a caloric goal of 60–70 % of the calculated requirement) versus target feeding (caloric goal of 90–100 % of the calculated requirement) with either IIT or CIT in critically ill patients [26]. The study found no difference between the two groups in 28-day mortality (18 % vs. 23 %, *p* = 0.34) [26]. However, hospital mortality was lower in the permissive underfeeding compared with the target-feeding group (30 % vs. 43 %, *p* = 0.04) [26]. All the patients enrolled in the original two trials were included in this study. In brief, these patients were ≥18 year-old with expected ICU length of stay(LOS) of >48 h. Patients who were pregnant, had do-not-resuscitate status within 24 h of admission, were terminally ill or admitted to the ICU after cardiac arrest, seizures, liver transplantation or burn injury were excluded. This study was approved by the Ministry of National Guard Health Affairs (NGHA) /King Abdullah International Medical Research Center (KAIMRC) Institutional Review Board. The consent for the present analysis was waived because of the observational nature of the study.

### Aspirin therapy

Data of aspirin therapy in the ICU were collected from the Pharmaceutical Care database and was matched and combined with the original clinical database. Aspirin therapy was either as a continuation of a pre-ICU prescription or a newly prescribed medication in the ICU per the treating team discretion, such as for patients admitted with stroke or acute coronary syndrome.

### Data collection

The following data were retrieved from the two original studies: age, gender, Acute Physiology and Chronic Health Evaluation (APACHE II) score [27], sequential organ failure assessment (SOFA) score [28], chronic co-morbidities (chronic liver, chronic cardiovascular, chronic respiratory, chronic renal and chronic immunocompromised) as defined by the APACHE II system, history of diabetes mellitus, admission diagnosis category (respiratory, cardiovascular, neurological, other medical, non-operative trauma and post-operative), mechanical ventilation (MV), sepsis and severe sepsis on admission. We also documented the statin use, bilirubin level, serum creatinine level, admission Glasgow Coma Scale (GCS) score [28], vasopressor use, The arterial partial pressure of O_2_ and the fraction of inspired oxygen (PaO_2_/FiO_2_) ratio, international normalization ratio (INR), and platelet count.

### Outcomes

The primary outcomes were all-cause ICU mortality and hospital mortality. Secondary endpoints included the development of severe sepsis during the ICU stay, ICU and hospital LOS, and MV duration.

### Statistical analysis

Statistical analysis was carried out using the Statistical Analysis Software (SAS, release 9, SAS Institute, Cary, NC, 2005). We compared patients who received aspirin during their ICU admission (aspirin group) to those who did not (non-aspirin group). Categorical data were presented as frequency with percent, whereas continuous variables were presented as mean with and standard deviation. Baseline characteristics and outcome variables were compared using the Chi-square test for nominal data and the Student *t* -test for continuous data. Due to the non-random allocation to aspirin use and significant differences between the groups in terms of baseline characteristics, a propensity score was calculated using the variables related to the aspirin exposure and outcome. These variables were age, sex, diabetes history, admission category, APACHE II score, chronic respiratory disease, chronic renal disease, chronic immunosuppression, creatinine, PaO_2_/FiO_2_ ratio, platelets, and statins Furthermore, the association between aspirin therapy each outcome was assessed by performing multivariate logistic regression analysis adjusting for the generated propensity score. Additionally, we carried out stratified analyses by age, gender, admission category, APACHE II, history of diabetes, the presence of chronic cardiac disease, vasopressor therapy, sepsis, severe sepsis and septic shock, creatinine, platelet count, bilirubin, INR, GCS, ICU LOS stay, MV, PaO_2_/FiO_2_ ratio, and the type of statins to detect any effect modification of the association between aspirin therapy and different outcome measures based on any of these factors. The medians of the continuous variables were used for categorization. The results of the multivariate analysis were presented as adjusted odds ratios (aOR) or point estimate with the 95 % confidence intervals (CI). We tested for interaction to assess effect modification between subgroups. Statistical significance was defined as *p* value ≤ 0.05.

## Results

### Patients characteristics

Of the 763 patients enrolled in the study, 154 (20.2 %) received aspirin at a dose of 81 mg daily during their ICU stay. Table 1 presents the comparison in baseline characteristics between aspirin and non-aspirin group. Patients who received aspirin were older, more likely to be males, had higher APACHE II scores, more likely to be on statins, with higher serum creatinine, more likely to be diabetics, with chronic cardiac, renal and respiratory illnesses and also with with lower platelets. When adjusted for propensity score, all these differences became insignificant.

**Table 1:** Baseline characteristics of the aspirin and non-aspirin therapy groups

Variable	Aspirin *N* = 154	Non-aspirin *N* = 609	Crude *P* value	PS adjusted * P*-Value
Age (yrs) mean ± SD	65 ± 16.5	48.8 ± 21.7	<0.0001	0.88
Gender, n (%)				
Female	56 (36.4)	152 (25)	0.005	0.97
Male	98 (63.6)	457 (75.1)		
Diabetes, n (%)	102 (66.2)	201 (33.8)	0.0001	0.22
Admission category, n (%)				
Non-operative	145 (94.2)	495 (81.3)	0.0001	0.21
Post-operative	9 (5.8)	114 (18.7)		
APACHE II, mean ± SD	26.5 ± 7.2	22.9 ± 8.2	<0.0001	0.67
Mechanical ventilation, n (%)	135 (87.7)	548 (90)	0.40	0.93
SOFA on day 1, mean ± SD	9.3 ± 3.3	9.2 ± 3.5	0.82	0.52
Sepsis, n (%)	47 (30.5)	147 (24.1)	0.104	0.68
Chronic respiratory disease, n (%)	43 (27.9)	114 (18.7)	0.011	0.19
Chronic cardiac disease n (%)	76 (49.4)	81 (13.3)	<0.0001	0.86
Chronic liver disease n (%)	9 (5.8)	45 (7.4)	0.50	0.54
Chronic immunosuppression n (%)	11 (7.1)	55 (9.1)	0.45	0.30
Chronic renal disease n%	38 (24.7)	65 (10.7)	0.0001	0.43
GCS, mean ± SD	9.0 ± 4.2	8.6 ± 4.1	0.23	0.70
Vasopressor, n (%)	106 (68.8)	390 (64.1)	0.26	0.36
PaO_2_/FiO_2_ ratio, mean ± SD	200 ± 98	221 ± 119	0.04	0.88
Statin use n%	74 (48.1)	33 (5.4)	<0.0001	0.83
Bilirubin (μmol/L), mean ± SD^a^	31.2 ± 69.4	36.1 ± 70	0.50	0.26
Creatinine (μmol/L), mean ± SD^a^	199.9 ± 171.2	148.8 ± 146.5	0.0002	0.15
INR, mean ± SD	1.5 ± 0.8	1.5 ± 0.9	0.73	0.26
Platelet, x 10^9^/L mean ± SD	193.1 ± 121	247.9 ± 133.3	<0.0001	0.048

*P*-values are provided for the differences between the two groups significant before and after propensity score adjustment

*APACHE* Acute physiology and chronic health evaluation, *GCS* Glasgow coma scale, *INR* International normalized ratio, *PS* propensity score, *SOFA* Sequential Organ Failure Assessment

^a^To convert to conventional units in mg/dL, divide by 88.4 for creatinine and 17.1 for bilirubin

### Outcomes

Table 2 summarized the association between aspirin therapy and mortality using multivariate analysis adjusted for propensity score. Aspirin therapy was not associated with ICU mortality (adjusted OR 1.18, 95 % CI 0.69–2.02, *P* = 0.55) nor with hospital mortality (adjusted OR 0.95, 95 % CI 0.61–1.50, *P* = 0.82). On the other hand, aspirin therapy was associated with higher risk of ICU-acquired severe sepsis (adjusted OR 1.70, 95 % CI 1.08–2.70, *P* = 0.02), increased MV duration (adjusted point estimate 2.7 days, 95 % CI 0.51–4.9, *P* = 0.02), and ICU length of stay (adjusted point estimate 2.67 days, 95 % CI 0.38–4.10, *P* = 0.02).

**Table 2:** Outcomes results of the aspirin and non-aspirin therapy groups

	Variable	Aspirin *N* = 154	Non-aspirin *N* = 609	Crude odds ratio			Adjusted odds ratio		
				OR	95 % CI	*P* value	OR	95 % CI	*P* value
Categorical	ICU mortality n (%)	32 (20.8)	95 (15.6)	1.42	0.91,2.22	0.12	1.18	0.69, 2.02	0.55
	Hospital mortality n (%)	62 (40.3)	180 (29.6)	1.61	1.11,2.32	0.01	0.95	0.61, 1.50	0.82
	Sepsis n (%)	56 (36.4)	256 (42.04)	0.79	0.55,1.36	0.20	1.18	0.76, 1.84	0.45
	Severe sepsis n (%)	51 (33.1)	182 (29.9)	1.16	0.80,1.70	0.44	1.70	1.08, 2.70	0.02
	28 day mortality n (%)	19 (30.7)	31 (17.4)	2.09	1.08,4.07	0.02	1.41	0.65, 3.05	0.39
	180 day mortality n (%)	50 (34.1)	128 (22.7)	1.75	1.18, 2.60	0.005	0.72	0.43, 1.21	0.22
Continuous				point estimate	95 % CI	*P* value	point estimate	95 % CI	*P* value
	MV duration (days)	11.17 ± 14.92	9.60 ± 9.05	1.58	−0.28, 3.44	0.09	2.7	0.51, 4.90	0.02
	ICU LOS (days)	12.4 ± 15.2	10.8 ± 9.5	1.7	−0.27, 3.58	0.09	2.67	0.38,4.96	0.02
	Hospital LOS (days)	65.6 ± 107.2	58.4 ± 81.7	7.22	−8.3, 22.7	0.36	12.5	−5.90, 30.96	0.18

*CI* Confidence interval, *MV* Mechanical ventilation, *OR* Odds ratio

Table 3 shows the association between aspirin therapy and all-cause hospital mortality in several subgroups of patients. Aspirin therapy was not associated with lower hospital mortality among any of the studied subgroups.

**Table 3:** Adjusted hospital mortality risk for subgroups

Variable	Adjusted odds ratio	95 % confidence interval	*P* value for interaction
All patients	0.95	0.60, 1.49	0.82
Age ≤ 58	0.76	0.25, 1.99	0.12
Age > 58	0.99	0.60, 1.67	
Males	0.65	0.35, 1.21	0.26
Females	1.59	0.78, 3.24	
Diabetes, yes	0.81	0.46, 1.42	0.002
no	1.09	0.52, 2.29	
APACHE ≤ 23	0.37	0.13, 1.11	0.96
APACHE > 23	1.14	0.67, 1.94	
Sepsis present	0.89	0.41,1.93	0.16
Sepsis absent	1.04	0.59, 1.82	
Severe sepsis present	1.23	0.58, 2.60	0.12
Severe sepsis absent	0.70	0.39, 1.27	
Non operative	0.95	0.60, 1.50	0.88
Post-operative	0.34	0.02, 4.43	
Chronic Respiratory disease			
Yes	1.18	0.54, 2.60	0.39
No	0.80	0.45, 1.42	
Chronic Cardiac disease			
Yes	0.92	0.47, 1.80	0.21
No	0.75	0.39, 1.43	
Chronic Renal disease			
Yes	0.81	0.30, 2.20	0.55
No	0.96	0.57, 1.60	
Chronic liver disease			
Yes	0.28	0.03, 2.24	0.21
No	1.05	0.66, 1.66	
Chronic immunosuppression			
Yes	0.76	0.15, 3.73	0.14
No	1.003	0.63, 1.61	
Platelets × 10^9^/L ≤ 184	0.71	0.35, 1.46	0.73
Platelets × 10^9^/L > 184	1.34	0.74, 2.44	
Bilirubin (μmol/l) ≤ 16	0.76	0.36, 1.62	0.16
Bilirubin (μmol/l) > 16	0.87	0.40, 1.90	
Creatinine (μmol/l) ≤ 102	0.97	0.41, 2.28	0.39
Creatinine (μmol/l) > 102	0.83	0.49, 1.42	
Statin users	1.17	0.50, 2.74	0.74
Statin non-users	0.75	0.42, 1.32	
PaO_2_/FiO_2_ ratio >200	0.43	0.19,0.97	0.14
PaO_2_/FiO_2_ ratio <200	1.56	0.88,2.79	

^a^Adjusted for propensity score which was calculated from age, sex, diabetes, admission category, APACHE, chronic respiratory disease, chronic renal disease, chronic immunosuppression, creatinine, PaO_2_/FiO_2_ ratio, platelets, and statin use

## Discussion

The present study showed that the use of aspirin in critically ill patients was not associated with lower mortality, but rather with increased morbidity.

Various retrospective clinical studies have shown that low-dose aspirin was associated with reduced mortality. Winning et al. conducted a single center retrospective cohort study on 615 critically ill patients, 25 % of whom were receiving antiplatelet drugs (aspirin or clopidogrel; 129 received aspirin in a dose of < 160 mg) and observed that patients on anti-platelets had lower ICU mortality (OR 0.19, 95 %, CI 0.12–0.33) on multivariable analysis [29]. The same investigators conducted a study on patients with community acquired pneumonia and found a decrease in the length of hospital stay in association with aspirin therapy [30]. Moreover, a large cohort study of ICU patients (*n* = 7,945) showed that low-dose aspirin was associated with reduced mortality among patients with systemic inflammatory response syndrome and sepsis although the risk of renal injury increased [31]. On the other hand, Valerio-Rojas et al. performed a retrospective cohort study and found that antiplatelet therapy was not associated with decrease in hospital mortality or ICU mortality [32]. The current study found no statically significant difference between the two groups in the ICU, hospital mortality after adjusting for the propensity to receive aspirin therapy. Rather, our study shows aspirin use is associated with higher risk of developing severe sepsis during ICU stay and an increase in the duration of mechanical ventilation and ICU length of stay.

Aspirin use has been studied for its possible effect in reducing the risk of acute lung injury (ALI). Erlich et al. found that pre-hospitalization antiplatelet therapy was associated with a reduced incidence of ALI (OR 0.34, 95 % CI 0.13–0.88) [33]. Another observational study had similar findings [32]. Another ICU cohort study found that aspirin potentiated the effect of statins in reducing ALI and sepsis [34]. More recently, in a secondary analysis of the Validating Acute Lung Injury Markers for Diagnosis study, Chen et al. found that prehospital aspirin use was associated with a decreased risk of acute respiratory distress syndrome (OR 0.66, 95 % CI, 0.46–0.94) [35]. However, Kor et al. conducted a large multicenter observational study that included 20 ICUs in the US and two ICUs in Turkey failed to confirm the beneficial effect of aspirin on ALI after adjusting for propensity score to receive aspirin therapy [36]. Our study did not examine the association with the incidence with ALI. However, it showed higher risk of ICU-associated severe sepsis. In addition, it showed that aspirin therapy was not associated with lower mortality in patients with ALI (OR 1.56, 95 % CI 0.88–2.79).

Aspirin has multiple effects in critically ill patients. As antiplatelet drugs irreversibly inhibit platelet function, they hinder their activation and surface expression of adhesion molecules like selectins and GPIIb IIIa receptors which are key in the formulation of microvascular thrombus [37]. Once microvascular thrombosis is formed, it causes ischemia that contributes to tissue injury and multiple organ dysfunction syndrome [38, 39]. Also, platelet inactivation attenuates the secretion of inflammatory mediators [7, 9], depresses their interaction with immune cells [40] and hence modulates the adverse effects associated with the inflammatory reaction [41]. Additionally, aspirin stimulates the synthesis of 15-epi-lipoxin A4, in which it increases the synthesis of nitric oxide through endothelial nitric oxide synthase and inducible nitric oxide synthase [42]. Nitric oxide inhibits the interactions between leucocytes and endothelial cells, decreasing poly-morph neutrophil recruitment [43]. Another putative effect of aspirin is the inhibition of the nuclear factor kappa-B [44], which plays an essential role in immune and inflammatory responses [45]. The lack of beneficial effect of aspirin may be a reflection of the interaction on these multiple pathways, where a possibly positive effect may be negated by a negative effect.

Our study should be interpreted in light of its strengths and limitations. The strengths of our study include being a nested cohort study within randomized controlled trials with prospective data collection and all the patients on the treatment group were on aspirin 81 mg. Yet, our study has several limitations, which includes its post-hoc design with the concern of unmeasured bias and confounders. To overcome this concern, we performed adjusted analysis using the propensity score. Other limitations include its monocenter nature, and the lack of data on the duration of aspirin therapy prior to ICU admission, cause of aspirin prescription and data on other antiplatelet agents, platelet transfusion, and aspirin side effects. Additionally, we have no data on bleeding risk or the development of ALI.

## Conclusions

Our study show that continuing aspirin therapy during ICU stay was not associated with reduction in ICU or hospital mortality in critically ill patients, but rather with an increased morbidity. This adds to the conflicting data and confirms the need for randomized, controlled trials to verify the relationship between aspirin therapy and the outcomes of critically ill patients. Currently, there is an ongoing trial of aspirin 200 mg once daily for 14 days compared with placebo in adult patients with severe sepsis and/or septic shock (NCT01784159). Furthermore, another multicenter randomized clinical trial is evaluating aspirin use in preventing ARDS (NCT01504867). We hope that these ongoing clinical trials will provide clear answers on the role of aspirin therapy in critical illness.

## Abbreviations

ALI: Acute lung injury
ARDS: Acute respiratory distress syndrome
MOF: Multiple organ failure
APACHE II: Acute physiology and chronic health evaluation
GCS: Glasgow coma scale
INR: International normalized ratio
PS: Propensity score

## References

[1] Levi M (2005). Platelets in sepsis. Hematology.

[2] Vincent JL, Yagushi A, Pradier O (2002). Platelet function in sepsis. Crit Care Med.

[3] Katz JN, Kolappa KP, Becker RC (2011). Beyond thrombosis: the versatile platelet in critical illness. Chest.

[4] Nguyen TC, Carcillo JA (2006). Bench-to-bedside review: thrombocytopenia-associated multiple organ failure--a newly appreciated syndrome in the critically ill. Crit Care.

[5] Levi M, Lowenberg EC (2008). Thrombocytopenia in critically ill patients. Semin Thromb Hemost.

[6] Flad HD, Brandt E (2010). Platelet-derived chemokines: pathophysiology and therapeutic aspects. Cell Mol Life Sci.

[7] Weyrich AS, Zimmerman GA (2004). Platelets: signaling cells in the immune continuum. Trends Immunol.

[8] Steinhubl SR (2007). Platelets as mediators of inflammation. Hematol Oncol Clin North Am.

[9] Gear AR, Camerini D (2003). Platelet chemokines and chemokine receptors: linking hemostasis, inflammation, and host defense. Microcirculation.

[10] Semple JW, Freedman J (2010). Platelets and innate immunity. Cell Mol Life Sci.

[11] Smyth SS, McEver RP, Weyrich AS, Morrell CN, Hoffman MR, Arepally GM (2009). Platelet functions beyond hemostasis. J Thromb Haemost.

[12] Peerschke EI, Yin W, Ghebrehiwet B (2008). Platelet mediated complement activation. Adv Exp Med Biol.

[13] Peerschke EI, Yin W, Alpert DR, Roubey RA, Salmon JE, Ghebrehiwet B (2009). Serum complement activation on heterologous platelets is associated with arterial thrombosis in patients with systemic lupus erythematosus and antiphospholipid antibodies. Lupus.

[14] Prandoni P, Ghirarduzzi A, Prins MH, Pengo V, Davidson BL, Sorensen H (2006). Venous thromboembolism and the risk of subsequent symptomatic atherosclerosis. J Thromb Haemost.

[15] Wiesner J, Vilcinskas A (2010). Antimicrobial peptides: the ancient arm of the human immune system. Virulence.

[16] Nguyen LT, Kwakman PH, Chan DI, Liu Z, de Boer L, Zaat SA (2011). Exploring platelet chemokine antimicrobial activity: nuclear magnetic resonance backbone dynamics of NAP-2 and TC-1. Antimicrob Agents Chemother.

[17] Clark SR, Ma AC, Tavener SA, McDonald B, Goodarzi Z, Kelly MM (2007). Platelet TLR4 activates neutrophil extracellular traps to ensnare bacteria in septic blood. Nat Med.

[18] Setzer F, Oberle V, Blass M, Moller E, Russwurm S, Deigner HP (2006). Platelet-derived microvesicles induce differential gene expression in monocytic cells: a DNA microarray study. Platelets.

[19] Ridker PM, Cushman M, Stampfer MJ, Tracy RP, Hennekens CH (1997). Inflammation, aspirin, and the risk of cardiovascular disease in apparently healthy men. N Engl J Med.

[20] Steinhubl SR, Badimon JJ, Bhatt DL, Herbert JM, Luscher TF (2007). Clinical evidence for anti-inflammatory effects of antiplatelet therapy in patients with atherothrombotic disease. Vasc Med.

[21] Graff J, Harder S, Wahl O, Scheuermann EH, Gossmann J (2005). Anti-inflammatory effects of clopidogrel intake in renal transplant patients: effects on platelet-leukocyte interactions, platelet CD40 ligand expression, and proinflammatory biomarkers. Clin Pharmacol Ther.

[22] Muhlestein JB (2010). Effect of antiplatelet therapy on inflammatory markers in atherothrombotic patients. Thromb Haemost.

[23] Ikonomidis I, Andreotti F, Economou E, Stefanadis C, Toutouzas P, Nihoyannopoulos P (1999). Increased proinflammatory cytokines in patients with chronic stable angina and their reduction by aspirin. Circulation.

[24] Arabi Y, Alshimemeri A, Taher S (2006). Weekend and weeknight admissions have the same outcome of weekday admissions to an intensive care unit with onsite intensivist coverage. Crit Care Med.

[25] Arabi YM, Dabbagh OC, Tamim HM, Al-Shimemeri AA, Memish ZA, Haddad SH, et al. Intensive versus conventional insulin therapy: a randomized controlled trial in medical and surgical critically ill patients. Crit Care Med. 2008;36(12):3190–7. Trial registration: Current Controlled Trials registry ISRCTN07413772. 10.1097/CCM.0b013e31818f21aa 18936702

[26] Arabi YM, Tamim HM, Dhar GS, Al-Dawood A, Al-Sultan M, Sakkijha MH, et al. Permissive underfeeding and intensive insulin therapy in critically ill patients: a randomized controlled trial. Am J Clin Nutr. 2011;93(3):569–77. Trial registration: Current Controlled Trials registry: ISRCTN96294863. 10.3945/ajcn.110.005074 21270385

[27] Knaus WA, Draper EA, Wagner DP, Zimmerman JE. APACHE II: a severity of disease classification system. Crit Care Med. 1985;13(10):818–29. 3928249

[28] Ferreira FL, Bota DP, Bross A, Melot C, Vincent JL (2001). Serial evaluation of the SOFA score to predict outcome in critically ill patients. JAMA.

[29] Winning J, Neumann J, Kohl M, Claus RA, Reinhart K, Bauer M (2010). Antiplatelet drugs and outcome in mixed admissions to an intensive care unit. Crit Care Med.

[30] Winning J, Reichel J, Eisenhut Y, Hamacher J, Kohl M, Deigner HP (2009). Anti-platelet drugs and outcome in severe infection: clinical impact and underlying mechanisms. Platelets.

[31] Eisen DP, Reid D, McBryde ES (2012). Acetyl salicylic acid usage and mortality in critically ill patients with the systemic inflammatory response syndrome and sepsis. Crit Care Med.

[32] Valerio-Rojas JC, Jaffer IJ, Kor DJ, Gajic O, Cartin-Ceba R (2013). Outcomes of severe sepsis and septic shock patients on chronic antiplatelet treatment: a historical cohort study. Critical care research and practice.

[33] Erlich JM, Talmor DS, Cartin-Ceba R, Gajic O, Kor DJ (2011). Prehospitalization antiplatelet therapy is associated with a reduced incidence of acute lung injury: a population-based cohort study. Chest.

[34] O’Neal HR, Koyama T, Koehler EA, Siew E, Curtis BR, Fremont RD (2011). Prehospital statin and aspirin use and the prevalence of severe sepsis and acute lung injury/acute respiratory distress syndrome. Crit Care Med.

[35] Chen W, Janz DR, Bastarache JA, May AK, O’Neal HR, Bernard GR (2015). Prehospital aspirin use is associated with reduced risk of acute respiratory distress syndrome in critically ill patients: a propensity-adjusted analysis. Crit Care Med.

[36] Kor DJ, Erlich J, Gong MN, Malinchoc M, Carter RE, Gajic O (2011). Association of prehospitalization aspirin therapy and acute lung injury: results of a multicenter international observational study of at-risk patients. Crit Care Med.

[37] McEver RP (2002). Selectins: lectins that initiate cell adhesion under flow. Curr Opin Cell Biol.

[38] Gando S (2010). Microvascular thrombosis and multiple organ dysfunction syndrome. Crit Care Med.

[39] Semeraro N, Ammollo CT, Semeraro F, Colucci M (2012). Sepsis, thrombosis and organ dysfunction. Thromb Res.

[40] Evangelista V, Manarini S, Dell’Elba G, Martelli N, Napoleone E, Di Santo A (2005). Clopidogrel inhibits platelet-leukocyte adhesion and platelet-dependent leukocyte activation. Thromb Haemost.

[41] Garcia CC, Guabiraba R, Soriani FM, Teixeira MM (2010). The development of anti-inflammatory drugs for infectious diseases. Discov Med.

[42] Paul-Clark MJ, Van Cao T, Moradi-Bidhendi N, Cooper D, Gilroy DW (2004). 15-epi-lipoxin A4-mediated induction of nitric oxide explains how aspirin inhibits acute inflammation. J Exp Med.

[43] Morris T, Stables M, Hobbs A, de Souza P, Colville-Nash P, Warner T (2009). Effects of low-dose aspirin on acute inflammatory responses in humans. J Immunol.

[44] Yin MJ, Yamamoto Y, Gaynor RB (1998). The anti-inflammatory agents aspirin and salicylate inhibit the activity of I(kappa)B kinase-beta. Nature.

[45] Abraham E (2003). Nuclear factor-kappaB and its role in sepsis-associated organ failure. J Infect Dis.

